# The role of isolation rooms, facemasks and intensified hand hygiene in the prevention of nosocomial COVID-19 transmission in a pulmonary clinical setting

**DOI:** 10.1186/s40249-020-00725-z

**Published:** 2020-07-23

**Authors:** Gu-Qin Zhang, Hua-Qin Pan, Xing-Xing Hu, Shao-Jun He, Yi-Fei Chen, Chao-Jie Wei, Lan Ni, Li-Ping Zhang, Zhen-Shun Cheng, Jiong Yang

**Affiliations:** 1 Department of Respiratory and Critical Care Medicine, Zhongnan Hospital of Wuhan University, 169 Eastlake Road, Wuhan, 430071 China; 2 Department of Critical Care Medicine, Zhongnan Hospital of Wuhan University, Wuhan, 430071 China; 3 Department of Social Medical Development, Zhongnan Hospital of Wuhan University, Wuhan, 430071 China

**Keywords:** COVID-19, SARS-CoV-2, Isolation room, Facemask, Hand hygiene

## Abstract

From December 25, 2019 to January 31, 2020, 33 cases of the coronavirus disease 2019 (COVID-19) were identified in the Department of Respiratory and Critical Care Medicine of Zhongnan Hospital of Wuhan University, China, yet none of the affiliated HCWs was infected. Here we analyzed the infection control measures used in three different departments in the Zhongnan Hospital of Wuhan University and correlated the measures with the corresponding infection data of HCWs affiliated with these departments. We found that three infection control measures, namely the isolation of the presumed positive patients, the use of facemasks and intensified hand hygiene play important roles in preventing nosocomial transmission of COVID-19.

## Background

In December 2019, a series of acute respiratory cases of unknown etiology SARS-CoV-2emerged [1]. This disease was later named coronavirus disease 2019 (COVID-19) caused by severe acute respiratory syndrome coronavirus 2 (SARS-CoV-2, also called 2019-nCoV). As of April 23, 2020, 82 804 people in total have been confirmed infection and 5.6% of the confirmed cases have died in China. Additionally, COVID-19 cases have been reported in more than 200 countries outside of China [2]. The World Health Organization (WHO) has declared the COVID-19 a public health emergency of international concern [3]. Accumulating evidences have demonstrated the human-to-human transmission of SARS-CoV-2 [4–7]. In the early stage of epidemic, especially before the SARS-CoV-2 was identified and announced, the causative agent and the transmission route were unclear, many healthcare workers (HCWs) were infected with SARS-CoV-2 due to close contact with COVID-19 patients and inadequate protective measures. As of February 24, 2020, 3387 COVID-19 cases among HCWs from 476 medical institutions in China have been identified with COVID-19, 90% of these cases coming from Hubei Province [8], therefore causing a profound social and economic burden to the medical system. Such nosocomial transmission of COVID-19 has been observed in several departments in the Zhongnan Hospital of Wuhan University located in Wuhan, Hubei. Strikingly, in our institution, despite having 33 patients diagnosed with COVID-19 in Department of Respiratory and Critical Care Medicine, none of the 73 HCWs were infected, indicative of successful preventive methods used by our department. In this study, we compared the infection control measures along with the infection data of HCWs in the Department of Respiratory and Critical Care Medicine with that of two departments from the same hospital that showed positive cases for nosocomial transmission.

## Methods

### Internal workflow of COVID-19 controlling in Zhongnan Hospital of Wuhan University

From early January 2020, a group of medical doctors was selected to form a consultation board for the identification and controlling of “pneumonia of unknown etiology”. All departments of our hospital were required to screen all the inpatients. Patients with respiratory symptoms would first receive a chest computerized tomography (CT) scan. If features representing viral pneumonia were detected through the CT scan, the pharyngeal swab specimens would be collected from the patients and then be subjected to reverse-transcriptase polymerase-chain-reaction (RT-PCR) assay to test for SARS-CoV-2. Patients with positive test results would be immediately transferred to specialized infectious disease hospitals or admitted to the isolation ward in our hospital. The wards where the positive patients first appeared would undergo terminal cleaning after the patients were transferred. From early January, the HCWs with respiratory symptoms or with history of close contacting with COVID-19 patient would receive a chest CT scan and RT-PCR assay for pharyngeal swab specimens to test for SARS-CoV-2. In late January, all the HCWs were scanned by chest CT scan and RT-PCR assay for pharyngeal swab specimens to test for SARS-CoV-2, suspected or confirmed cases were admitted to the isolation ward in our hospital.

### Data sources

Cases were diagnosed based on the Diagnosis and Treatment Guidelines for COVID-19 (Trial seventh version) that issued by the National Health Committee of the People’s Republic of China [9]. All confirmed cases in this study were defined as a positive result in high-throughput sequencing for bronchoalveolar lavage fluid or real-time RT-PCR assay for pharyngeal swab specimens. The severity of COVID-19 was defined based on the international guidelines for community-acquired pneumonia [10].

### Clinical settings in the Department of Respiratory and Critical Care Medicine

During the time of the outbreak, our department consisted of a 37-bed inpatient unit (unit A), a 74-bed inpatient unit (unit B), and a 20-bed respiratory and critical care unit (unit C). Unit A was located at floor 17, whereas unit B and unit C were located in the same corridor at floor 16 but were separated by a gate. In unit A and unit B, our policy was to hospitalize each non-severe patient with respiratory disease. All family members and friends were allowed to visit patients in any time. In unit C, only severe patients were admitted and only one family member or friend was allowed to visit the patient in prescript time.

### Infection control measures in the Department of Respiratory and Critical Care Medicine

On Dec 25, 2019, a 39-year-old male (patient 1) with 5 day of fever and cough was admitted to unit A in the Department of Respiratory and Critical Care Medicine with the diagnosis of “pneumonia”. He showed multifocal patchy ground-glass opacities on chest CT scans, especially around the peripheral parts of the lungs, which were compatible with changes seen in viral pneumonia. His pharyngeal sample was negative for common respiratory virus including Influenza A, Influenza B, and avian influenza A H7N9 virus (H7N9). On Dec 28, 2019, a 21-year-old female (patient 2) with 6 days of fever and cough was admitted in unit B. Similarly, she showed multifocal patchy ground-glass opacities around the peripheral parts of the lungs on CT scan, and her pharyngeal sample was tested negative for common respiratory virus. On January 1, 2020, the bronchoalveolar lavage fluid (BALF) of patient 1 was collected and used for High-throughput sequencing, and the result on January 2, 2020 revealed the identification of a variant coronavirus. An emergency staff meeting was called and several decisions were taken as shown in Table 1.

**Table 1:** List of prevention actions against nosocomial transmission of SARS-CoV-2 in the Department of Respiratory and Critical Care Medicine

➢ Except the patient 1, all other patients in unit A were transferred to unit B. ➢ Unit A was then assigned as the primary unit for suspected cases of COVID-19. ➢ Patient 2 was transferred to unit A in a single-bed hospital room.	
➢ All inpatients were screened daily and the suspected cases were immediately transferred to unit A in individual single-bed hospital rooms.	
➢ All patients in unit A were asked to wear surgical masks and quarantined in their rooms, and visits were temporarily suspended in unit A.	
➢ Strict personal protective measures including N95 respirator, facemask, gloves, surgical cap, and isolation gown were required for HCWs in unit A.	
➢ All HCWs in unit A were reminded of the need for extreme vigilance with regard to hand hygiene protocols including the use of antiseptic soap or alcoholic hand rub, especially before and after contacting the patients or their surroundings.	
➢ All communal activities were suspended in unit A.	
➢ In unit B and unit C, the inpatients were hospitalized and treated following the standard routine, and the HCWs in these units were equipped with conventional protective measure the surgical mask and followed standard hand hygiene protocols.	

*COVID-19* Coronavirus disease 2019, *SARS-CoV-2* Severe acute respiratory syndrome coronavirus 2, *HCWs* Health care workers

### Infection control measures in the Departments of Hepatobiliary surgery and neurology

In the Departments of Hepatobiliary Surgery and Neurology, all the nurses and part of the doctors wore single-use medical mask when on duty and most HCWs followed the WHO’s My 5 Moments for hand hygiene protocol before the first affiliated COVID-19 patient was diagnosed. In the Department of Hepatobiliary Surgery, the patient was diagnosed as COVID-19 6 days after admission and immediately transferred to isolation ward in our hospital on the same day. In the Department of Neurology, two patients with acute cerebral infarction were diagnosed with COVID-19 five days after admission. Given their severe medical conditions, these patients were admitted to the Intensive Care Unit of Neurology (NCU), which contains 10 isolation bed in one hospital room. The COVID-19 patients were transferred to bed 1 and bed 2, respectively. Concomitantly, the patients originally on bed 3 to bed 5 were transferred to bed 6 to bed 10 for distancing purpose. Screens were also used between bed 1, 2, and 3 for more effective separation. After the first COVID-19 patient was diagnosed, all the HCWs in the Departments of Hepatobiliary Surgery and Neurology wore surgical mask when on duty and all the HCWs followed the WHO’s My 5 Moments for hand hygiene protocols. All communal activities were suspended in these departments.

## Results

### Clinical characters of COVID-19 patients associated with occupational exposure to HCWs

The risk of HCWs with exposure to COVID-19 are largely determined by the number and the length of stay of the COVID-19 patients in a given healthcare environment. Additionally, delayed diagnose of COVID-19, particularly in the patients with atypical symptoms, often results in increased exposure risk among HCWs. Moreover, severe COVID-19 patients likely show higher potency of disease dissemination as compared with non-severe cases and may contribute to the exposure risk of the HCWs who interact with those patients during medical care processes [11, 12]. Therefore, to assess the exposure risk of HCWs, we compared the number, the length of stay, the admission symptoms and the disease severity of COVID-19 patients in three departments.

As of January 31, 2020, a total of 33 patients was diagnosed with COVID-19 in the Department of Respiratory and Critical Care Medicine. The median age of the patients was 50-year-old (range: 21–88 years), and 18 patients (54.5%) were male. The most common admission symptoms were fever (84.8%), cough (45.5%), fatigue (45.5%), muscle ache (30.3%), and dyspnea (12.1%). The less common symptoms were palpitations (9.1%), stomachache (3%), diarrhea (3%), chest pain (3%), and vomit (3%). Until January 31, 2020, of the 33 patients, 19 (57.6%) cases were non-severe and 14 (42.4%) cases were severe. Twenty-six (78.8%) were transferred to the isolation wards in our hospital or the infectious disease hospital, five (15.2%) were recovered and discharged, and two (6.1%) died.

In the Department of Hepatobiliary Surgery, one patient with the admission symptom of stomachache was diagnosed as COVID-19 six days after admission and immediately transferred to isolation ward in our hospital on the same day. In the Department of Neurology, two patients with acute cerebral infarction were diagnosed with COVID-19 5 days after admission, one with fatigue and the other with diarrhea on admission (Table 2), the two cases were severe.

**Table 2:** The clinical features of patients with COVID-19 in three different departments

	Department		
	Respiratory (*n* = 33)	Hepatobiliary Surgery (*n* = 1)	Neurology (*n* = 2)
Age, Median (range)years	50 (21–88)	65 (65–65)	56 (48–64)
Gender, *n* (%)			
Male	18 (54.5)	0 (0)	2 (100)
Female	15 (45.5)	1 (100)	0 (00
Admission symptom, *n* (%)			
Fever	28 (84.8)	0 (0)	0 (0)
Cough	15 (45.5)	0 (0)	0 (0)
Fatigue	15 (45.5)	0 (0)	1 (50)
Muscle ache	10 (30.3)	0 (0)	0 (0)
Dyspnea	4 (12.1)	0 (0)	0 (0)
Palpitations	3 (9.1)	0 (0)	0 (0)
Dizziness	2 (6.1)	0 (0)	1 (50)
Diarrhea	1 (3)	0 (0)	0 (0)
Chest pain	1 (3)	0 (0)	0 (0)
Vomit	1 (3)	0 (0)	0 (0)
Stomachache	1 (3)	1 (100)	0 (0)
Hospital days, Median (range)	4 (1–14)	6 (6–6)	10 (10–10)
Non-severe cases, *n* (%)	19 (57.6)	1 (100)	0
Severe cases, *n* (%)	14 (42.4)	0 (0)	2 (100)
Outcome, *n* (%) (up to January 31, 2020)			
Discharge	5 (15.2)	0 (0)	0 (0)
Deceased	2 (6.1)	0 (0)	0 (0)
Transferred	26 (78.8)	1 (100)	0 (0)
In hospital	0	0 (0)	2 (100)

### Infection data of HCWs

As of January 31, 2020, all the HCWs were screened for COVID-19 by both chest CT and RT-PCR assay for SARS-CoV-2 with pharyngeal swab specimens. None of the HCWs in Department of Respiratory and Critical Care Medicine were infected. In the Department of Hepatobiliary Surgery, only one patient was confirmed with COVID-19 and she did not receive surgery due to her COVID-19 positive status. However, nine doctors and one nurse were confirmed infected with SARS-CoV-2 with one other nurse suspected of infection in that department **(**Table 3**)**. We further inquired the exposure history of the infected HCWs to identify potential reasons for the infection. The first symptomatic doctor reported that he did not wear mask when performing body examination of the patient. Additionally, one of the infected nurses reported that she cared for the patient before the patient was confirmed infection. Despite that the other infected HCWs did not have a history of direct contact with the COVID-19 patients, it was reported that all HCWs of the department joined a 2-hour-long meeting three days before the patient was diagnosed. The first symptomatic doctor showed fever two days after the meeting and was diagnosed with COVID-19 on the same day, while the other eight doctors and one nurse showed symptoms of fever or cough and were confirmed infection later. Such direct exposure mediated COVID-19 infection has also been shown in the Department of Neurology, where one doctor who performed physical examination of the patient and one nurse who cared for the patient were confirmed infection up to January 31, 2020 **(**Table 3). All the infected HCWs have fully recovered now.

**Table 3:** The infection data of HCWs in three different departments

	Respiratory	Hepatobiliary surgery	Neurology
**COVID-19 patients** (con/sus)	23/10	1/0	2/0
**HCWs**			
Total	73	74	90
Infected			
Doctors (*n*)	14	25	28
Con/sus	0/0	9/0	1/0
Nurse (*n*)	59	49	62
Con/sus	0/0	1/1	1/0
Percentage of infection (%)	0	14.9	2.2

*HCWs* Health care workers, *con/sus* Confirmed/suspected cases;

## Discussion

In this study, we compared the quarantine strategies used for COVID-19 patients, the screening process for HCWs, the infection control measures used by HCWs, and the infection data of HCWs across three departments of Zhongnan Hospital of Wuhan University. We found that the early isolation of suspected patients, use of surgical facemasks or N95 respirators, and intensified hand hygiene contribute to the prevention of nosocomial transmission of COVID-19.

Most COVID-19 patients presented typical respiratory symptoms including fever and cough, whereas several patients showed atypical symptoms [13, 14]. This is represented by the patient with stomachache from the Department of Hepatobiliary Surgery, and the patients with fatigue and diarrhea in the Department of Neurology. Because of their atypical symptoms, several HCWs had been contacted with the patient without adequate protective measures before the patients being confirmed of COVID-19, resulting in increased infection rate among HCWs in these two departments. This indicates that early rapid identification is a critical first step for preventing nosocomial COVID-19 transmission, and that more vigilance for patients with atypical respiratory symptoms is needed. Additionally, we implanted daily screening for all patients in Department of Respiratory and Critical Care Medicine for prompt identification of potentially contagious patients. This was accompanied with quarantine of the suspected patients along with a strict control of the visit policy. Due to these actions, none of other patient or HCWs in Department of Respiratory and Critical Care Medicine was infected, suggesting that early isolation of all potentially contagious is likely the most efficient practice to control the transmission of COVID-19. Indeed, WHO has also recommended isolation of the patients with suspected SARS-CoV-2 infection [15] and isolation has been widely applied in controlling COVID-19 epidemic in China and worldwide and has been confirmed effective.

The emergence of novel respiratory pathogens, such as SARS-CoV, pandemic H1N1 influenza (H1N1), and the current SARS-CoV-2 has emphasized the vulnerability of HCWs to respiratory infections. Thus, the individual protective equipment is critical for decreasing the occupational risk of respiratory infection in HCWs. WHO has recommended HCWs to wear medical masks when care for suspected or confirmed COVID-19 patients and promoted the use of the FFP2 type mask or N95 respirators only for aerosol-generating procedures [15]. Meta-analyses of observational studies have provided evidence of the protective effects of the surgical masks and N95 respirators against SARS [16]. However, the impacts of these protective measures in preventing HWCs from nosocomial transmission of COVID-19 remain unclear. In our study, the HCWs in unit A who cared for the suspected patients were required to wear N95 respirators, whereas the HCWs in unit B and C wore surgical masks. None of the HCWs in Department of Respiratory and Critical Care Medicine was infected, suggesting the N95 respirators provide effective protections against SARS-CoV-2. In December 2019, all the HCWs in Department of Respiratory and Critical Care Medicine wore surgical masks. Many of them interacted with the patient 1 and 2, but none were infected, indicative of adequate protective effect of surgical masks against SARS-CoV-2. In contrast, we found that all the nurse and part of the doctors in the Departments of Hepatobiliary Surgery and Neurology, only wore single-use medical masks instead of surgical masks on duty, and many of them were infected by the COVID-19 patients. This data suggests insufficient protective role of single-use medical mask against SARS-CoV-2. Taken together, we suggest all the HCWs wear surgical masks or N95 respirators when in contact with suspicious patients.

Hand-contact is a common transmission mode in infectious respiratory diseases. We applied the WHO’s My 5 Moments for hand hygiene approach before performing any clinical procedures, before and after touching patients and their surroundings, and after exposing to body fluid [17]. Previous studies have showed that HCWs who washed their hands while caring for SARS patients were less likely to be infected compared to those who did not [18]. In our study, HCWs in other departments who did not well follow the hand hygiene approach were more likely to develop COVID-19. This suggest that all the HCWs in the whole hospital need to improve the compliance to hand hygiene rate and should wear at least surgical mask or higher level of protective measures should be equipped if any putative COVID-19 cases appear in the department.

In this study, we showed that 10 HCWs were infected in the Department of Hepatobiliary Surgery despite that only one doctor and one nurse directly cared for the COVID-19 patient. However, we found that all the infected HCWs joined a meeting before the patient was diagnosed. Therefore, this nosocomial transmission event likely happened during the meeting, when the infected doctor and nurse have not developed any obvious symptoms. This suggests that there was likely pre-symptomatic transmission of the SARS-COV-2 to other HCWs in the meeting. Thus, reducing people gathering and keeping social distance, especially among HCWs, can serve as very important preventive measures for controlling the nosocomial transmission of SARS-CoV-2.

## Conclusions

Our findings indicate that the isolation rooms, the facemasks, and intensified hand hygiene seemed to prevent nosocomial transmission of SARS-CoV-2. These nonpharmaceutical interventions are important for preventing nosocomial transmission of pandemic respiratory diseases.

## Data Availability

The datasets used and/or analyzed during the current study are available from the corresponding author on reasonable request.

## Abbreviations

COVID-19: Coronavirus disease 2019
SARS-CoV-2: Severe acute respiratory syndrome coronavirus 2
HCWs: Health care workers
WHO: World Health Organization
BALF: Bronchoalveolar lavage fluid
SARS: Severe acute respiratory syndrome
SARS-CoV: Severe acute respiratory syndrome-associated coronavirus
pH1N1: Pandemic H1N1 influenza
Jan: January
Dec: December
